# Genetic signals of artificial and natural dispersal linked to colonization of South America by non‐native Chinook salmon (*Oncorhynchus tshawytscha*)

**DOI:** 10.1002/ece3.4036

**Published:** 2018-05-24

**Authors:** Daniel Gomez‐Uchida, Diego Cañas‐Rojas, Carla M. Riva‐Rossi, Javier E. Ciancio, Miguel A. Pascual, Billy Ernst, Eduardo Aedo, Selim S. Musleh, Francisca Valenzuela‐Aguayo, Thomas P. Quinn, James E. Seeb, Lisa W. Seeb

**Affiliations:** ^1^ Genomics in Ecology, Evolution and Conservation Lab (GEECLAB) Department of Zoology Facultad de Ciencias Naturales y Oceanográficas Universidad de Concepción Concepción Chile; ^2^ Núcleo Milenio INVASAL Concepción Chile; ^3^ Instituto de Diversidad y Evolución Austral IDEAUS‐CONICET Centro Nacional Patagónico Puerto Madryn Argentina; ^4^ Centro para el estudio de Sistemas Marinos CESIMAR‐CONICET Centro Nacional Patagónico Puerto Madryn Argentina; ^5^ Instituto Patagónico para el estudio de Ecosistemas Continentales IPEEC‐CONICET Centro Nacional Patagónico Puerto Madryn Argentina; ^6^ Department of Oceanography Universidad de Concepción Concepción Chile; ^7^ Facultad de Ciencias Naturales y Oceanográficas Universidad de Concepción Concepción Chile; ^8^ Centro Trapananda Universidad Austral de Chile Coyhaique Chile; ^9^ School of Aquatic and Fishery Sciences University of Washington Seattle WA USA; ^10^ Present address: Department of Aquatic Systems Faculty of Environmental Sciences and EULA‐Centre Universidad de Concepción Concepción Chile

**Keywords:** Argentina, Chile, genetic stock identification, individual assignment, invasion genetics, Pacific salmon

## Abstract

Genetics data have provided unprecedented insights into evolutionary aspects of colonization by non‐native populations. Yet, our understanding of how *artificial* (human‐mediated) and *natural* dispersal pathways of non‐native individuals influence genetic metrics, evolution of genetic structure, and admixture remains elusive. We capitalize on the widespread colonization of Chinook salmon *Oncorhynchus tshawytscha* in South America, mediated by both dispersal pathways, to address these issues using data from a panel of polymorphic SNPs. First, genetic diversity and the number of effective breeders (*N*_b_) were higher among *artificial* than *natural* populations. Contemporary gene flow was common between adjacent *artificial* and *natural* and adjacent *natural* populations, but uncommon between geographically distant populations. Second, genetic structure revealed four distinct clusters throughout the Chinook salmon distributional range with varying levels of genetic connectivity. Isolation by distance resulted from weak differentiation between adjacent *artificial* and *natural* and between *natural* populations, with strong differentiation between distant Pacific Ocean and Atlantic Ocean populations, which experienced strong genetic drift. Third, genetic mixture analyses revealed the presence of at least six donor geographic regions from North America, some of which likely hybridized as a result of multiple introductions. Relative propagule pressure or the proportion of Chinook salmon propagules introduced from various geographic regions according to government records significantly influenced genetic mixtures for two of three *artificial* populations. Our findings support a model of colonization in which high‐diversity *artificial* populations established first; some of these populations exhibited significant admixture resulting from propagule pressure. Low‐diversity *natural* populations were likely subsequently founded from a reduced number of individuals.

## 1 INTRODUCTION

Invasion biology has historically benefited from a partnership with population genetics to clarify evolutionary aspects of the establishment and spread of colonizing species (Allendorf & Lundquist, 2009; Baker & Stebbins, 1965; Barrett, 2015). Such interplay has motivated many genetics studies to identify the geographic origin of non‐native populations, assess postcolonization gains or losses in genetic diversity, and illuminate population connectivity and genetic structure to provide strategies for control (Dlugosch & Parker, 2008; Roman & Darling, 2007; Sakai et al., 2001). Yet, there are many unresolved questions on how *artificial* and *natural* dispersal pathways differentially influence the population genetics of colonizing populations. The former relates to human‐mediated releases or propagule pressure, intentional and unintentional, of non‐native individuals into the receiving environment; the latter relates to spread of non‐native individuals from established populations via gene flow. Populations founded via *artificial* dispersal may involve multiple introductions with expected increases in genetic diversity (Consuegra, Phillips, Gajardo, & de Leaniz, 2011; Kolbe et al., 2004; Simberloff, 2009), whereas *natural* populations may originate via founder effects and dispersal from established populations, exhibiting decreased genetic diversity (Kawamura et al., 2010; Kinziger, Nakamoto, Anderson, & Harvey, 2011; Rollins et al., 2013). However, little is known about how these two dispersal pathways of invasion differentially influence (i) genetic diversity and demography, namely the annual effective number of breeders (*N*_b_), (ii) spatial patterns of genetic structure, and (iii) the degree of genetic admixture among non‐native populations.

Pacific salmon species (genus *Oncorhynchus*) native to the Northern Hemisphere are important subjects in ecology and evolutionary biology because they exhibit diverse life histories (Quinn, 2005; Stearns & Hendry, 2004), and also because they have been successfully introduced around the world for commercial and recreational fisheries and aquaculture (Crawford & Muir, 2008). Introductions of Pacific salmon into the Southern Hemisphere, especially New Zealand and South America, have provided unique research opportunities to investigate the relative roles of propagule pressure, preadaptations, phenotypic plasticity, and low ecosystem resistance in explaining invasions (Arismendi et al., 2014; Pascual et al., 2007; Quinn, Kinnison, & Unwin, 2001). Several genetics studies in South America have ascertained the origin of donor (native) salmonid populations (Ciancio, Riva‐Rossi, Pascual, Anderson, & Garza, 2015; Riva‐Rossi, Lessa, & Pascual, 2004; Riva‐Rossi et al., 2012). Other studies have shown significant gains in genetic diversity among non‐native populations that may be important for invasion success (Correa & Moran, 2017; Di Prinzio, Rossi, Ciancio, Garza, & Casaux, 2015; Narum et al., 2017). Yet, answers to fundamental questions of how salmonids have established and spread throughout South America, a phenomenon that is invariably related to how and where they have been propagated (*artificial* vs. *natural*), are lacking. Anadromous salmonids that spawn in freshwater but feed in the ocean as adults are especially appropriate to study as they can rapidly colonize unoccupied habitats by dispersal. They can become quickly established, extending their distribution at their native (Hendry, Castric, Kinnison, & Quinn, 2004; Quinn, 2005) and non‐native ranges (Quinn et al., 2001).

Chinook salmon (*O. tshawytscha*) is the largest‐bodied Pacific salmon species, recognized for its importance for recreational, commercial, and subsistence fisheries in North America and Asia. Chinook salmon were also repeatedly introduced to South America via several government‐sponsored and private initiatives during most of the twentieth century, both in Chile and in Argentina, to develop recreational and commercial fisheries as well as net‐pen aquaculture (Basulto, 2003; Pascual & Ciancio, 2007). Several studies indicate that successful introductions of Chinook salmon to South America, with adults returning in large numbers, occurred following sea‐ranching experiments in the Lake (X Region) and Magallanes (XII Region) districts in Chile at the end of 1970s and beginning of 1980s (Correa & Gross, 2008; Niklitschek & Toledo, 2011; Riva‐Rossi et al., 2012; Soto, Arismendi, Di Prinzio, & Jara, 2007). Many other populations have formed beyond initial and well‐documented stocking sites, suggesting that Chinook salmon in South America comprise both *artificial* and *natural* populations (Table 1). Past and recent genetics and genomics studies have identified multiple donor populations of Chinook salmon that likely interbred and now coexist among Pacific Ocean and Atlantic Ocean basins (Correa & Moran, 2017; Narum et al., 2017; Riva‐Rossi et al., 2012). Both individual assignment and genetic analysis of population mixtures (McKinney, Seeb, & Seeb, 2017) have greatly assisted tracking the geographic origin of donor Chinook salmon populations to various sites in South America (Ciancio et al., 2015; Correa & Moran, 2017; Di Prinzio et al., 2015). However, we lack a clear understanding on how genetic diversity, dispersal, and genetic admixture are linked to *artificial* and *natural* dispersal pathways of this species from its distributional range in South America. This is crucial to understand how colonization by non‐native Chinook salmon has unfolded in less than 40 years (Correa & Gross, 2008; Riva‐Rossi et al., 2012).

**Table 1:** Study sites of non‐native Chinook salmon in South America, their reported donor sources, and predicted dispersal pathways

Ocean	Country, Region	Basin	River (Code)	Donor populations and geographic origin^a^^,^ b ^,^ c ^,^ d ^,^ e	Dispersal pathway	Population remarks^a^^,^ b ^,^ c ^,^ e ^,^ f
Pacific Ocean	Chile, Araucania	Toltén	Allipén (ALP)	Unknown	Artificial	Release of unknown broodstock to Estero El Membrillo (inlet tributary of Allipén River) from failed hatchery operation in 1995. Returning adults to the Toltén River estuary in 2000. Around 12,000 returning adults reported during austral spring and summer in 2014‐2015
	Chile, Los Lagos	Petrohué	Petrohué (PET)	Lower Columbia River, University of Washington broodstock, New Zealand, Oregon	Artificial	Combination of sea‐ranching (1979–1989) and releases of aquaculture fish (1990–1999) near the estuary located in the inner sea of Chiloe. Hundreds to thousands of returning adults every year to spawn during austral summer and fall
	Chile, Los Lagos	Pichicolo	Pichicolo (PIC)	University of Washington broodstock	Artificial	Hatchery population from University of Washington broodstock. The original source was Green River Hatchery in South Puget Sound, Washington
	Chile, Aysén	Aysén	Cobarde (COB)	Vancouver (British Columbia)	Artificial	Net‐pen aquaculture releases at inner fjords of Aysén District
	Chile, Aysén	Baker	Vargas (VAR)	Unknown	Natural	Dispersal of propagules from an established population further north at Cobarde River (COB). Unknown number of returning adults
	Chile, Magallanes	Serrano	Serrano (SER)	Unknown	Natural	Dispersal from a nearby established population at Prat River (PRA). Unknown number of returning adults
	Chile, Magallanes	Prat	Prat (PRA)	Lower Columbia River, Oregon, University of Washington broodstock	Artificial	Successful sea‐ranching experiments during 1982–1986. Most likely the donor population of propagules for Atlantic Ocean populations (SAC, CAT). No information on numbers of returning adults
Atlantic Ocean	Argentina, Santa Cruz	Santa Cruz	Santa Cruz (SAC)	Lower Columbia River via Pacific Ocean basins	Natural	Main river stem of Santa Cruz basin. Founded by natural dispersal from established Pacific Ocean populations, possibly Prat River (PRA). Unknown number of returning adults
	Argentina, Santa Cruz	Santa Cruz	Caterina (CAT)	Lower Columbia River via Pacific Ocean basins	Natural	Tributary to Santa Cruz River (SAC) and one of the main spawning grounds. Unknown number of spawning adults

a Correa and Gross (2008).

b Niklitschek and Toledo (2011).

c Riva‐Rossi et al. (2012).

d Chile's Undersecretariat of Fisheries, unpublished data.

e Ciancio et al. (2015).

f Gomez‐Uchida et al. (2016).

**Figure 1:**
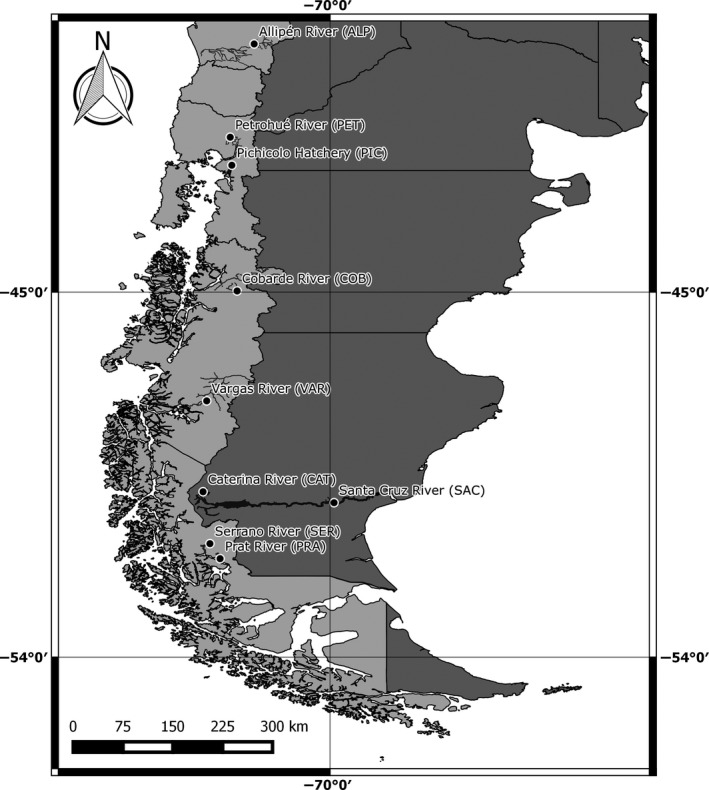
Distribution of non‐native Chinook salmon sampled locations (circles) in South America. Light gray, Pacific Ocean basins; dark gray, Atlantic Ocean basins

Here we analyzed nine Chinook salmon collections taken from *artificial* and *natural* populations (defined *a priori*) through their South American distribution, including Pacific Ocean and Atlantic Ocean basins (Figure 1), using a panel of 172 polymorphic SNPs. These markers were developed from an ascertainment panel of wild and hatchery populations from the native range in North America (Warheit, Seeb, Templin, & Seeb, 2012) and have proven to be extremely informative among non‐native populations. We used individual‐ and population‐based inference, introduction records, and a baseline of genetic information from donor populations to address three goals in relation to the colonization history of Chinook salmon in South America. First, we quantified genetic diversity, contemporary dispersal, and *N*_b_ among populations to test the prediction that *artificial* populations should harbor more genetic diversity and have larger estimates of *N*_b_ than *natural* populations. We also tested whether contemporary dispersal was evident from *artificial* to *natural* populations, assuming the former were established first and the latter subsequently founded. Second, we evaluated genetic divergence and tested for genetic isolation by distance. We predicted that genetic differentiation may be weaker between adjacent *artificial* and *natural* populations assuming ongoing gene flow, but stronger between distant pairs, especially if genetic drift strongly influenced *natural* populations. Third, we inferred which donor (native) populations contributed to establishment of non‐native Chinook salmon using analyses of genetic mixtures. We predicted whether genetic mixtures were consistent with relative propagule pressure or the proportion of Chinook salmon propagules introduced from various geographic regions according to historical government records.

## 2 MATERIALS AND METHODS

### 2.1 Sampling

We covered the entire distributional range of Chinook salmon among Pacific Ocean and Atlantic Ocean basins in South America by supplementing archived samples of seven populations (Riva‐Rossi et al., 2012) with contemporary collections of two populations. Archived samples were collected during 2005–2009 and included Estero Pichicolo (PIC), a hatchery population, and Pacific Ocean rivers Cobarde (COB), Vargas (VAR), Serrano (SER), and Prat (PRA). Atlantic Ocean rivers included Santa Cruz (SAC) and Caterina (CAT), a small tributary of Argentino Lake that feeds SAC, the main stem of the basin (Figure 1). Contemporary samples were collected from freshly spawned carcasses at Petrohué River (PET) during May 2013 and from juvenile parr collected at Allipén River (ALP) during November 2014. Fin clips were either preserved dried in silica or in ethanol 95% for laboratory analyses.

### 2.2 SNP genotyping

Genomic DNA was isolated using a Macherey‐Nagel NucleoSpin^®^ Tissue kit (Düren, Germany) following the protocols from the manufacturer. Some isolates from Chinook salmon carcasses contained low concentrations of DNA (<10 ng/μl), and these generally yielded low‐quality genotypes that were excluded from analyses. DNA was screened for a suite of 191 SNPs (Table S1) chosen from a larger database (288 SNPs) developed to coordinate genomic resources available for improving Chinook salmon fisheries management (Warheit et al., 2012). Exploratory analyses showed that different suites of SNPs had similar information content and performed equally well for fisheries applications (Warheit et al., 2012). Genotyping was performed on Fluidigm^®^ 96.96 dynamic arrays under PCR conditions and concentrations recommended by Seeb et al. (2007), following a preamplification step by Smith et al. (2011).

### 2.3 SNP selection

We tested whether our data fit Hardy–Weinberg equilibrium (HWE) proportions or showed evidence of linkage disequilibrium (LD) using GENEPOP v4.2 (Raymond & Rousset, 1995b; Rousset, 2008) through 10,000 dememorization steps, 100 batches, and 5000 iterations per batch, using the complete enumeration method. SNPs that consistently departed from HWE proportions or were found in LD in more than half of locations were excluded from subsequent analyses.

### 2.4 Genetic diversity, *N*_b_, and gene flow

We estimated observed (*H*_O_) and expected heterozygosity (*H*_E_) for each collection using GENALEX (Peakall & Smouse, 2012) assuming populations defined *a priori*. Intrapopulation inbreeding coefficients (*f*) were calculated in FSTAT (Goudet, 2001) following Weir and Cockerham (1984). We implemented a permutational multivariate analysis of variance (PERMANOVA) from package *vegan* for R (R Core Team 2016) to test for differences in expected heterozygosity between *artificial* and *natural* populations.

The effective number of breeders per population (*N*_b_) was calculated in NeEstimator using the linkage disequilibrium (LD) approach (Do et al., 2014; Waples & Do, 2010). The method has the important advantage of requiring a single sample to providing unbiased estimates of contemporary *N*_b_ per brood year in age‐structured populations, including semelparous salmonids with variable age at maturity. We ran NeEstimator on these settings: (i) threshold values for minor allele frequencies dependent on sample sizes *n *≥* *25 or *n *<* *25, (ii) random mating system, and (iii) jackknifing to calculate 95% CIs (Waples & Do, 2010). We tested whether LD *N*_b_ differed between *artificial* and *natural* populations using a nonparametric Mann–Whitney test in R.

First‐generation immigrants were identified using GENECLASS 2.0 (Piry et al., 2004) to gauge contemporary gene flow between *artificial* and *natural* populations. We used Bayesian assignment of genotype likelihoods following Rannala and Mountain (1997). Genotypes were then ranked according to Paetkau, Slade, Burden, and Estoup (2004) in order to relate the likelihood of drawing them from the populations in which they were sampled with the maximum likelihood of such genotypes considering any of the study populations. The method assumes that all sources of migrants have been sampled. This may not be the case and “ghost” unsampled populations may influence these analyses, suggesting estimates of gene flow and identification of immigrants need to be interpreted with caution. We used 10,000 simulations and set type I error α = 0.01 to minimize erroneous identification of immigrants.

### 2.5 Genetic structure and isolation by distance

First, we used an individual‐based discriminant analysis of principal components (DAPC) (Jombart 2008) on multilocus genotypes to analyze genetic structure. Clustering of individuals was performed by maximizing their genetic proximity using the *k‐mean* algorithm. We varied *k* or the number of clusters between *k *=* *2 and *k *=* *9 and tested the significance of each *k* using the first 150 principal components and Bayesian information criterion.

Second, we estimated pairwise genetic divergence between populations using Weir and Cockerham (1984) *θ* estimator. Exact tests of population differentiation for the null hypothesis θ = 0 (Raymond & Rousset, 1995a) were applied using GENEPOP and 10,000 dememorization steps, 100 batches, and 1000 iterations per batch. We used Fisher's method in GENEPOP to combine exact tests per locus over multiple loci. We also tested whether pairwise genetic and geographic distances were significantly correlated using a simple Mantel test implemented in GENALEX. Separate tests and plots were conducted for pairwise *artificial*–*artificial*,* artificial*–*natural*, and *natural*–*natural* population comparisons to disentangle their relative roles in the correlation. Coastal distances (km) between population sampling sites were estimated using the tracking line option in QGIS (QGIS Development Team 2009).

### 2.6 Genetic mixtures and relative propagule pressure

#### 2.6.1 Baseline of donor populations

Potential donor populations were identified from a baseline of 41 native populations ranging from Alaska to California, each containing 48 individuals genotyped through a suite of 192 SNPs (Warheit et al., 2012). Statistical analyses nested native populations into 14 large geographic regions (Warheit et al., 2012). Geographic regions represent genetic lineages showing strong reproductive isolation, allowing accurate inference of the origin of individual fish (Moran et al., 2013; Seeb et al., 2007). Non‐native populations were thus matched to geographic regions, not individual populations, in order to minimize misassignment to genetically similar populations.

#### 2.6.2 Simulations

First, we tested for the accuracy afforded by baseline SNP genotypes among geographic regions to correctly assign simulated mixtures using ONCOR (Kalinowski, Manlove, & Taper, 2008) and the option 100% simulations. We simulated 200 mixtures of 100 individuals from each of 14 geographic regions following Anderson, Waples, and Kalinowski (2008). Second, we simulated hybrid classes (F1, F2, and backcrosses) in HYBRIDLAB (Nielsen, Bach, & Kotlicki, 2006) from randomly sampling 100 genotypes from each of two selected geographic regions. We explored whether true hybrids could be reliably assigned to their parental genotypes using mixture analysis, because mtDNA evidence suggests that substantial hybridization may have occurred between founding lineages in South America (Riva‐Rossi et al., 2012).

#### 2.6.3 Membership probabilities and mixture proportions to donor geographic regions

We performed mixture analysis in ONCOR to estimate mixture proportions of geographic regions using conditional maximum likelihood (Millar, 1987). We additionally estimated individual membership probabilities to each of 14 regions using Bayesian assignment (Rannala & Mountain, 1997) in ONCOR. Individual membership probabilities were represented as stacked bar plots in R, with colors depicting regions.

#### 2.6.4 Relative propagule pressure

We compared results from genetic mixtures to relative propagule pressure defined as the proportion of donor regions identified on government records. We surveyed information from three well‐documented basins impacted by captive breeding programs (stocking or aquaculture): Petrohue River (PET), Cobarde River (COB), and Prat River (PRA). Two primary sources of data were reviewed. First, we accessed databases belonging to Chile's Under Secretariat of Fisheries (SUBPESCA), Department of Aquaculture, to identify the geographic origin of Chinook salmon eggs importations for captive breeding taken place between 1977 and 2000, including geographic coordinates of stocking and farming operations. Introduction records from earlier periods were ignored because they likely failed to establish (Correa & Gross, 2008; Niklitschek & Toledo, 2011). Second, we accessed files from Chile's National Fisheries Service (SERNAPESCA) to verify which importations effectively took place. We multiplied propagule number (number of propagation events) by propagule mean size or frequency (average number of propagules per event) to estimate propagule pressure and their relative geographic contributions from various origins, often a city or state in North America with Chinook salmon hatchery facilities. Hatchery locations were matched to geographic regions based on geographic location (Warheit et al., 2012; Appendix 1). We then tested the hypothesis that present‐day genetic mixtures (as estimated via ONCOR; see above) were consistent with relative propagule pressure. We assessed deviations of observed from expected proportions using a Pearson chi‐square test in R, assuming such deviations follow a chi‐square distribution.

## 3 RESULTS

### 3.1 SNP selection

We successfully genotyped 342 Chinook salmon from nine locations (Table 2). Nine of 191 loci attempted were in LD in more than half of the locations and were removed, keeping the most informative following Gomez‐Uchida et al. (2011). Nine monomorphic loci and one locus that showed deviations from HWE proportions across locations were also removed (Table S1), leaving 172 SNPs for downstream analyses.

**Table 2:** Sample information and genetic statistics from non‐native Chinook salmon populations in South America (*n*: sample size, *H*_O_: observed heterozygosity, *H*_E_: expected heterozygosity, *f*: inbreeding coefficient, LD *N*_b_: linkage disequilibrium effective number of breeders)

Basin/River	Predicted dispersal	Sampling period	Life stage	*n*	*H*_O_	*H*_E_	*f*	LD *N*_b_ (95% CI)
Pacific Ocean								
ALP	Artificial	Spring 2014	Parr, Smolts	79	0.289	0.287	−0.007	375 (210–1046)
PET	Artificial	Fall 2013	Spawned carcasses	70	0.313	0.303	−0.025	230 (133–680)
PIC	Artificial	Summer 2010	Adults	25	0.325	0.294	−0.090	50 (32–105)
COB	Artificial	Summer 2005 and 2006Fall 2005 and 2006	Adults	36	0.273	0.279	0.014	99 (49–669)
VAR	Natural	Summer 2006	Adults	24	0.257	0.266	0.021	49 (39–66)
SER	Natural	Summer 2009	Adults	15	0.263	0.256	−0.033	44 (26–114)
PRA	Artificial	Summer 2006	Adults	30	0.289	0.302	0.042	28 (19–48)
Atlantic Ocean								
SAC	Natural	Spring 2005, 2008 and 2009; Winter 2010	Smolts	18	0.244	0.230	−0.056	20 (16–25)
CAT	Natural	Summer 2003 and 2008;Fall 2003 and 2004	Adults	45	0.208	0.207	−0.006	13 (9–19)

### 3.2 Genetic diversity, *N*_b_, and gene flow

**Figure 2:**
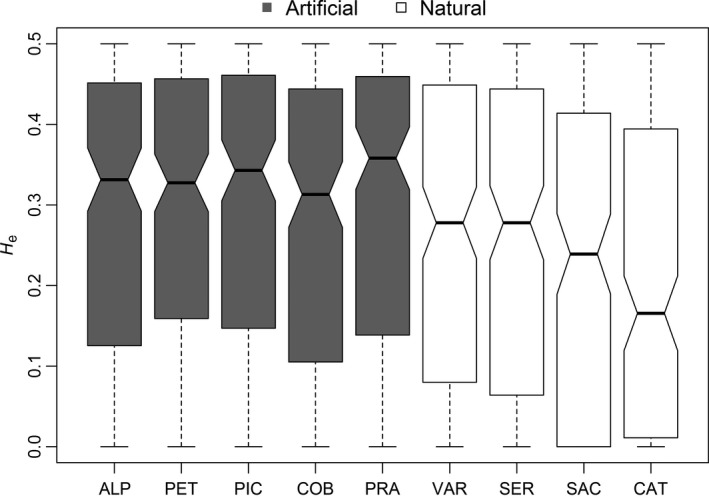
Boxplots of expected heterozygosity between artificially (*artificial*) and naturally dispersed (*natural*) non‐native Chinook salmon sampled from nine populations in South America. Abbreviations for river locations are as follows: ALP, Allipén River; PET, Petrohué River; PIC, Estero Pichicolo; COB, Cobarde River; VAR, Vargas River; SER, Serrano River; PRA, Prat River; SAC, Santa Cruz River; CAT, Caterina River

Values for *H*_O_ and *H*_E_ varied similarly across populations (Table 2); *H*_E_ fluctuated between 0.207 (CAT, Atlantic Ocean) and 0.303 (PET, Pacific Ocean). *Artificial* populations showed higher diversity than *natural* populations (PERMANOVA: *p* = .036; Figure 2). We found no significant inbreeding coefficients, consistent with fit of observed to expected HWE proportions. Estimates of LD *N*_b_ varied between 375 (ALP) and 13 (CAT; Table 2). *Artificial* populations had on average larger estimates of LD *N*_b_ than *natural* populations (Mann–Whitney test: *W* = 18, *p* = .031). *Artificial N*_b_ estimates averaged 156, whereas *natural N*_b_ estimates averaged 32. Neither *H*_E_ (Spearman's *r* = .41; *p* = .26) nor LD *N*_b_ was correlated to sample size (*r* = .57, *p* = .15), implying sampling bias failed to explain differences in *H*_E_ and LD *N*_b_ among populations.

We identified 14 first‐generation immigrants at the *p *<* *.01 threshold (Table 3). Gene flow between adjacent populations (10 of 14 immigrants) occurred from *artificial* to *natural* populations as predicted (COB to VAR) as well as in the opposite direction (VAR to PRA). We also observed dispersal among adjacent *natural* populations (SER and VAR; SAC and CAT). We observed additional long‐distance, bidirectional dispersal with four immigrants between COB in the Pacific Ocean and CAT in the Atlantic Ocean, two populations separated by nearly 4000 km of coastal distance. We found no evidence for connectivity between northern (ALP and PET) and southern populations (all the rest), other than one possible immigrant from PIC (a hatchery population) to PET.

**Table 3:** Number of first‐generation immigrants and their source populations identified using Bayesian individual assignment (dark colors = max values)

	Emigrating from								
Immigrating to	ALP	PIC	PET	COB	VAR	SER	PRA	SAC	CAT
ALP	–								
PIC		–							
PET		1	–						
COB				–					3
VAR				3	–				
SER					2	–			
PRA					2		–		
SAC								–	2
CAT				1					–

### 3.3 Genetic structure and isolation by distance

**Figure 3:**
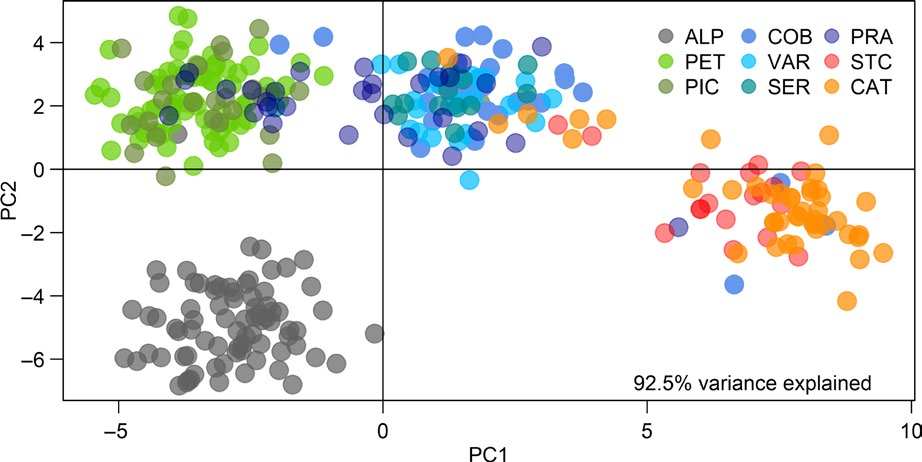
Discriminant analysis of principal components (DAPC) among Chinook salmon SNP multilocus genotypes from nine populations in South America. Abbreviations for river locations are as follows: ALP, Allipén River; PET, Petrohué River; PIC, Estero Pichicolo; COB, Cobarde River; VAR, Vargas River; SER, Serrano River; PRA, Prat River; SAC, Santa Cruz River; CAT, Caterina River

DAPC identified four clusters of Chinook salmon from South America: (1) ALP; (2) PET‐PIC; (3) COB‐VAR‐SER‐PRA; and (4) SAC‐CAT (Figure 3). Gene pools were discrete and consistent with *artificial* events in Pacific Ocean sites located north (ALP, PET‐PIC), even though admixture was evident between *artificial* Pacific Ocean sites located south (between COB and PRA). *Natural* populations from the Pacific Ocean (VAR and SER) clustered geographically close to *artificial* populations (COB and PRA). Atlantic Ocean sites SAC and CAT were the exception as they showed substantial divergence from all Pacific Ocean sites.

**Figure 4:**
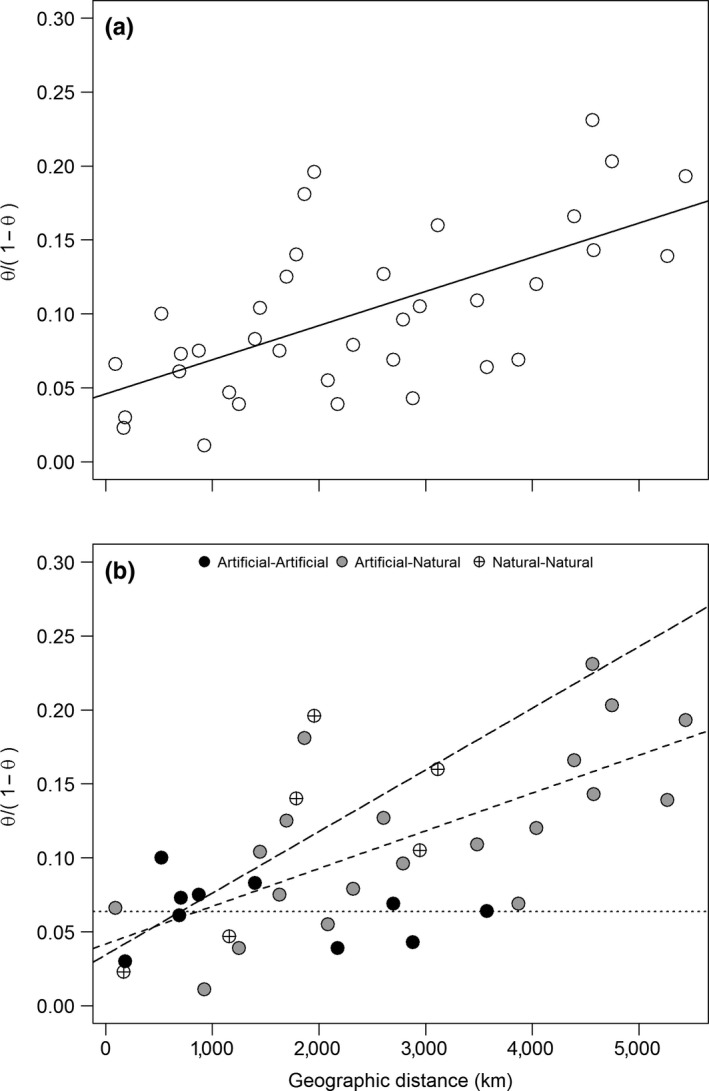
Scatterplot between linearized genetic and geographic distances among non‐native Chinook salmon populations from South America and best‐fit regression lines from two datasets: (a) pairwise comparisons among all nine populations and (b) pairwise comparisons between *artificial* populations (*artificial*–*artificial*; best‐fit, dotted line), between *artificial* and *natural* populations (*artificial*–*natural*; best‐fit, n‐dash line), and between *natural* populations (*natural*–*natural*; best‐fit, m‐dash line)

Genetic distance values fluctuated between *θ* = 0.011 (COB vs. VAR) and *θ* *= *0.231 (PIC vs. CAT) and were all significantly higher than 0, suggesting weak to strong spatial genetic structure (Table S2). A simple Mantel test revealed that *θ* and geographic distances were significantly correlated (*r *=* *.613, *p* < .001; Figure 4a). However, it became evident that the significance of this relationship was chiefly driven by pairwise comparisons between *artificial* and *natural* populations, and between *natural* populations. Comparisons between *artificial* populations showed no significant relationship between *θ* and geographic distances (*r *=* *.19, *p* > .10; Figure 4b).

### 3.4 Genetic mixtures and relative propagule pressure

#### 3.4.1 Baseline of donor populations and simulations

There was significant overlap between donor and non‐native suites of SNPs, although they were not identical. We found 127 SNPs in the non‐native populations that matched the donor population database (Table S1); only these were subsequently used for these analyses. First, simulated mixtures created from the baseline of native populations were correctly assigned to their geographic region in 99% or 100% of cases, strongly suggesting estimation of mixture proportions and individual assignment was highly accurate using 127 SNPs (Table S3). Second, F1, F2, and backcrosses simulated in HYBRIDLAB were reliably assigned to two selected parental geographic regions (*Oregon‐California Coast* and *Lower Columbia River‐Willamette*) with only 1 of 200 backcrossed individuals misassigned to a third, and closely related, parental geographic region (*Columbia River—Deschutes*: Figure S1).

#### 3.4.2 Membership probabilities and mixture proportions to donor geographic regions

Mixture analyses in ONCOR revealed six donor geographic regions of non‐native Chinook salmon (from north to south): (i) *Puget Sound—South British Columbia*, (ii) *Pacific Northwest—Washington Coastal*, (iii) *Lower Columbia River—Willamette*; (iv) *Columbia River—Deschutes*, (v) *Oregon‐California Coast*, and (vi) *California Central Valley* (Table 4). *Artificial* populations were composed of two or more geographic regions, whereas *natural* populations were composed of one geographic region. ALP showed the highest number of donor geographic regions with five, followed by PET with four geographic regions. Half of genotypes from ALP were assigned to *Oregon—California Coast*, whereas the majority of PET genotypes were assigned to *Puget Sound—South British Columbia*. The dominant geographic region in all remaining sites was *Lower Columbia River—Willamette*, including southern Pacific Ocean and Atlantic Ocean sites, with exception of PIC and PRA that had contributions from other geographic regions. PRA also showed nearly equal membership to *Puget Sound—South British Columbia* (43.2%) and *Lower Columbia River—Willamette* (56.9%). Individual Bayesian assignment was consistent with results above and further confirmed evidence of hybridization among donor populations as some individuals showed ancestry to two or more geographic regions (Figure S2).

**Table 4:** Mixture analysis among non‐native Chinook salmon populations with proportions assigned (and 95% CIs) to donor (native) geographic regions (sorted from north to south, presented from left to right)

	Puget Sound—South British Columbia	Pacific Northwest—Washington Coastal	Lower Columbia River—Willamette	Columbia River—Deschutes	Oregon‐California Coast	California Central Valley
ALP	0.093 (0.028, 0.204)	0.027 (0.000, 0.086)	0.364 (0.264, 0.625)	0.059 (0.007, 0.153)	0.457 (0.189, 0.512)	–
PET	0.903 (0.727, 0.971)	–	0.030 (0.000, 0.093)	–	0.022 (0.000, 0.117)	0.045 (0.000, 0.109)
PIC	0.861 (0.649, 0.989)	–	–	0.113 (0.000, 0.256)	–	0.027 (0.000, 0.145)
COB	0.028 (0.000, 0.083)	–	0.961 (0.875, 1.000)	–	0.012 (0.000, 0.089)	–
VAR	–	–	1.000 (0.874, 1.000)	–	–	–
SER	–	–	1.000 (0.803, 1.000)	–	–	–
PRA	0.432 (0.290, 0.598)	–	0.569 (0.401, 0.710)	–	–	–
SAC	–	–	1.000 (0.742, 1.000)	–	–	–
CAT	–	–	1.000 (0.982, 1.000)	–	–	–

#### 3.4.3 Relative propagule pressure

**Figure 5:**
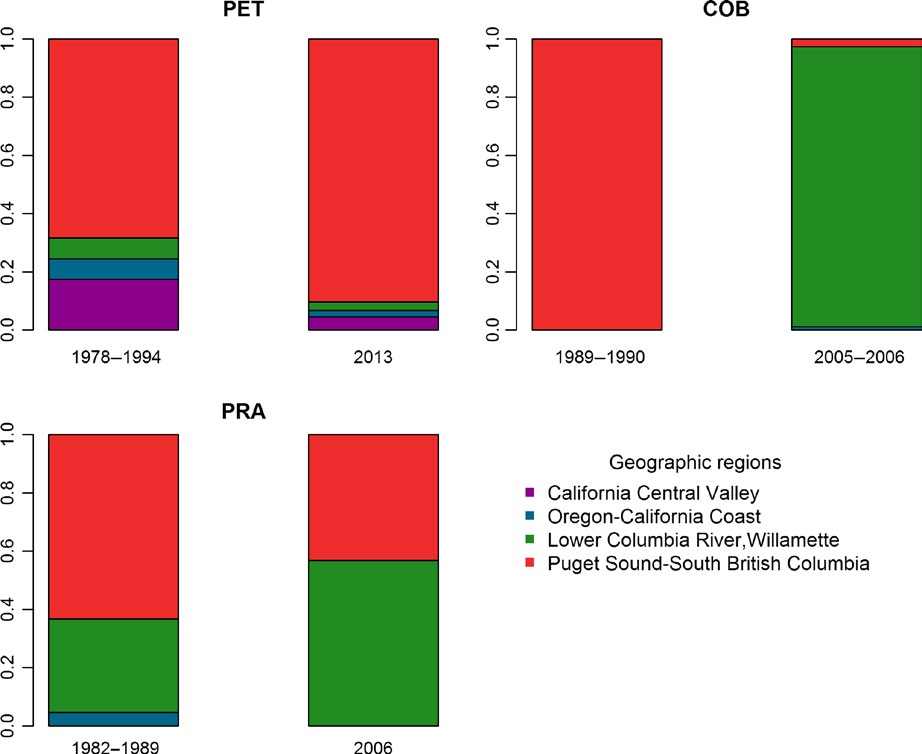
Stacked bars depicting relative propagule pressure by way of historical records compared to contemporary genetic mixtures for three non‐native Chinook salmon populations from South America. For each site, left bar indicates sources of propagules through time; right bar shows contemporary genetic mixture analysis and years of sampling, proportionally assigned to various geographic regions (i.e., native lineages of genetically similar populations) on PET, Petrohue River; COB, Cobarde River; PRA, Prat River

The highest propagule pressure invariably originated via Seattle in Washington, USA, for basins PET and PRA, and via Vancouver in British Columbia, Canada, for COB, with both locations of origin of propagules assigned to *Puget Sound‐South British Columbia* (Table 5). Introduction records further showed other locations in North America, including Lower Columbia River and Oregon hatcheries, and other countries such as New Zealand, which was only found among introductions to PET (Table 5). We found that genetic mixture proportions were consistent with relative propagule pressure proportions estimated for PET (Pearson χ^2^ = 0.223, *p* = .974) and PRA (Pearson χ^2^ = 0.302, *p* = .860: Figure 5). The exception was COB (Pearson χ^2^ = 40.13 *p* < .0001) for which genetic mixtures comprised higher than 95% genotypes from *Lower Columbia River‐Willamette*, yet 100% propagules were introduced via Vancouver in British Columbia, Canada, assigned to *Puget Sound‐South British Columbia* (Figure 5).

**Table 5:** Propagule pressure analyses for three *artificial* populations of Chinook salmon in South America

Population	Time period	City| State| Country of origin	Geographic region assigned	Propagule number	Propagule mean size	Source
Petrohue River (PET)	1978—1994	NA| NA| New Zealand	California Central Valley	1	2,500,000	Undersecretariat of Fisheries (unpublished)
		Lower Columbia River| Washington| USA	Lower Columbia River	3	336,667	Joyner (1980); Fundación‐Chile (1990), Undersecretariat of Fisheries (unpublished)
		NA| Oregon| USA	Oregon‐California Coast	1	1,000,000	Undersecretariat of Fisheries (unpublished)
		Seattle| Washington| USA	Puget Sound‐South British Columbia	14	696,786	Undersecretariat of Fisheries (unpublished)
						Undersecretariat of Fisheries (unpublished)
Cobarde River (COB)	1989—1990	Vancouver| British Columbia| Canada	Puget Sound‐South British Columbia	2	1,050,000	Undersecretariat of Fisheries (unpublished)
						Undersecretariat of Fisheries (unpublished)
Prat River (PRA)	1982—1989	NA| Oregon| USA	Oregon‐California Coast	1	50,000	Undersecretariat of Fisheries (unpublished)
		Lower Columbia River| Washington| USA	Lower Columbia River	1	340,000	Undersecretariat of Fisheries (unpublished)
		Seattle| Washington| USA	Puget Sound‐South British Columbia	3	291,667	Undersecretariat of Fisheries (unpublished)

NA, not available.

## 4 DISCUSSION

We used multilocus genotypes (from both non‐native and native and donor populations), historical records of introduction, and various statistical approaches to evaluate the roles of *artificial* and *natural* dispersal pathways of Chinook salmon (*Oncorhynchus tshawytscha*) in South America using a population genetics approach. Although Chinook salmon show strong philopatry, they also colonize new habitats via dispersal. Our genetic survey of nine non‐native populations covered nearly 5500 km and provided resolution across the distributional range to evaluate how colonization of this species has unfolded during almost four decades. Below we discuss how our findings informed and supported predictions regarding genetic diversity, the evolution of genetic structure, and population admixture.

### 4.1 Genetic diversity, *N*_b_, and gene flow

*Artificial* populations had on average higher genetic diversity and larger estimates of LD *N*_b_ than *natural* populations. This is consistent with two, nonmutually exclusive hypotheses. First, increased *H*_E_ among *artificial* populations may reflect admixture during captive breeding or *in situ* following introduction of multiple lineages (van Boheemen et al., 2017; Kolbe et al., 2004; Zalewski, Michalska‐Parda, Bartoszewicz, Kozakiewicz, & Brzezinski, 2010). Second, a decrease in *H*_E_ and LD *N*_b_ among *natural* populations may have occurred due to subsequent founder effects, which appeared especially pronounced among Chinook salmon populations that colonized Atlantic Ocean rivers. These possibly experienced further genetic drift subsequent to their colonization from populations previously established in Pacific Ocean basins (Ciancio et al., 2015; Riva‐Rossi et al., 2012). Mitochondrial gene diversity supports a similar result: D‐loop haplotype diversity among non‐native Chinook salmon in South America was higher among Pacific Ocean basins close to points of introduction than in so‐called peripheral populations such as CAT (Riva‐Rossi et al., 2012). Chinook salmon from Futaleufú River (not included in this study) also exhibited increased heterozygosity, consistent with their origin from net‐pen aquaculture (Di Prinzio et al., 2015).

Canales‐Aguirre et al. (2018) found that estimates of LD *N*_b_ were higher for non‐native rainbow trout populating a Patagonian lake with intensive trout aquaculture than for a lake where trout aquaculture has been prohibited by law. This highlights a possible role of continuous trout escapes and artificial dispersal on enlarging *N*_b_. Præbel, Gjelland, Salonen, and Amundsen (2013) found that introduced vendace *Coregonus albula* populations in a large European basin, some of which were part of a secondary expansion by natural dispersal, had a lower effective population size (a quantity related to *N*_b_) than originally stocked populations. Yet, our estimates of LD *N*_b_ need to be interpreted with caution due to some drawbacks. First, samples were taken in different years and comprised different life stages and thus applied to slightly different periods. Waples (2005) argued that estimates of LD *N*_b_ from adults (e.g., most samples from this study) sampled at year *i* apply to previous years, likely year *i–j* where *j* is the age at which adults reproduce. Estimates of LD *N*_b_ from juveniles (e.g., parr or smolts from populations ALP and SAC) sampled at year *i* apply to the same year (Waples, 2005). Second, most adult samples included one or two consecutive brood years, with exception of CAT that included multiple (pooled) brood years; also, smolt samples from SAC were pooled across 5 years. Estimates for these populations approach the effective population size (*N*_e_) rather than *N*_b_ as samples span one entire generation of Chinook salmon (Waples, 2005; Waples & Do, 2010).

Patterns of contemporary gene flow indicated that dispersal among adjacent populations occurred in all possible directions. We observed gene flow from *artificial* to *natural* populations (COB to VAR in the Pacific Ocean), suggesting that *artificial* populations were likely established first and *natural* populations were subsequently founded (VAR to PRA in the Pacific Ocean). We also observed gene flow between *natural* populations (VAR to SER in the Pacific Ocean; SAC to CAT in the Atlantic Ocean). Further, we found evidence for long‐distance dispersal between Pacific Ocean and Atlantic Ocean basins (COB to CAT and vice versa). Both strategies may be common among successful invasions (Wilson, Dormontt, Prentis, Lowe, & Richardson, 2009). Long‐distance dispersal in particular appears crucial for establishment and spread of invasive populations in both theoretical (Ibrahim, Nichols, & Hewitt, 1996; Shigesada, Kawasaki, & Takeda, 1995) and empirical studies spanning various taxa and environments [birds: da Silva, Eberhard, Wright, Avery, and Russello (2010); fishes: Bronnenhuber, Dufour, Higgs, and Heath (2011); invertebrates: Tobin and Blackburn (2008); plants: Puzey and Vallejo‐Marín (2014)]. Findings from Riva‐Rossi et al. (2012) also support the observation of long‐distance dispersal as mtDNA D‐loop sequences from Chinook salmon sampled at CAT in the Atlantic Ocean were closely related to COB in the Pacific Ocean.

Both adjacent and long‐distance dispersal strategies appear to be important for establishment and spread of Chinook salmon, similar to observations in other invasive fishes (Bronnenhuber et al., 2011). Unwin and Quinn (1993) reported straying rates 4–20% among non‐native Chinook salmon in New Zealand, with most occurring proximate to the tagging site. However, habitat variation causes salmon to stray selectively, so proximity is not the only factor influencing dispersal (Pascual & Quinn, 1994; Westley, Dittman, Ward, & Quinn, 2015). Additionally, the influence of oceanographic currents flowing southward along the southeastern Pacific Ocean (Cape Horn Current) and east into the Atlantic Ocean (West Wind Drift) may provide a directional component to dispersal from the Pacific Ocean to the Atlantic Ocean resulting in long‐distance gene flow (Becker, Pascual, & Basso, 2007; Ciancio et al., 2015; Riva‐Rossi et al., 2012).

### 4.2 Genetic structure and isolation by distance

DAPC identified three clusters consistent with independent stocking events among *artificial* populations in Pacific Ocean basins (e.g., ALP, PET, PRA), even though some amount of gene flow has occurred between *artificial* (COB, PRA) and *natural* (VAR, SER) populations, especially south of 45°S. Also, PRA was stocked with Chinook salmon that likely founded PET (Correa & Gross, 2008; Riva‐Rossi et al., 2012), which explains why PRA and PET genotypes partially overlap in DAPC analyses despite the fact that nearly 3,000 km separate the two populations. A fourth cluster comprised Chinook salmon from SAC and CAT, two *natural* Atlantic Ocean populations that showed strong differentiation (*θ* = 0.069–0.231; Table S2) from all Pacific Ocean basins. Results from two mitochondrial DNA studies (Becker et al., 2007; Riva‐Rossi et al., 2012) and another using biparental SNPs (Ciancio et al., 2015) failed to support successful stocking of Chinook salmon from California, United States, to Atlantic Ocean basins at the beginning of the twentieth century. On the contrary, those studies supported the origin of propagules from Pacific Ocean basins during more recent periods. Sea‐ranching operations that released juvenile Chinook salmon at PRA from 1982 to 1988 were likely the source of founders for Santa Cruz River (SAC), wherein spawning grounds are found at CAT.

The first records of Chinook salmon by anglers at CAT dated back to 1979–1984, supporting the Pacific Ocean origin hypothesis (Ciancio, Pascual, Lancelotti, Rossi, & Botto, 2005). A strong founder effect and subsequent genetic drift are possible explanations as to why SAC and CAT strongly diverged from Pacific Ocean basin sources. Yet, selection cannot be discounted as another explanation for divergence, especially among colonizing populations (Hanfling, 2007; Lee, 2002). Narum et al. (2017) examined adaptive genomic variation by comparing native and non‐native Chinook salmon sampled from Patagonia and found evidence for 118 outlier SNPs that deviated from neutral expectations (1%; 11,579 SNPs in total). Some of these outliers were linked to immune function, transposons, regulation of transcription, and histone acetylation (Narum et al., 2017). This is consistent with the concept of “favored founders”; they are not only a small subset of the source population, but a nonrandom, preadapted subset because they had to survive and reproduce to establish the population (Quinn et al., 2001). We therefore encourage further investigations on how adaptive divergence explains successful invasions, currently an active area of research on the use of thousands of SNPs to investigate genome signatures of selection (Puzey & Vallejo‐Marín, 2014; Vandepitte et al., 2014; White, Perkins, Heckel, & Searle, 2013).

Isolation by distance among invasive populations may evolve from genetic differentiation following geographic expansion from a single source (Herborg, Weetman, Van Oosterhout, & Hanfling, 2007; Kawamura et al., 2010; Kinnison, Bentzen, Unwin, & Quinn, 2002) or secondary contact between multiple introductions (Bifolchi, Picard, Lemaire, Cormier, & Pagano, 2010). None of these explanations seem to apply to Chinook salmon in South America. We hypothesize that this pattern has emerged from weak differentiation between adjacent *artificial* and *natural* populations as well as between *natural* populations, combined with strong differentiation between geographically distant populations influenced by genetic drift, namely Atlantic Ocean populations. This stems from the fact that *artificial* populations made no contribution to differentiation.

### 4.3 Genetic mixtures and relative propagule pressure

#### 4.3.1 Membership probabilities and mixture proportions to donor geographic regions

Genetic mixture analysis, a popular method used by management agencies in North America to ascertain the origin of unknown Pacific salmon (McKinney et al., 2017), has been increasingly adopted to address similar questions among invasive salmonids. Recent studies have analyzed pitfalls in the implementation of the method among non‐native populations, namely strong genetic drift (Ciancio et al., 2015) and hybridization (Correa & Moran, 2017) following introductions. Ciancio et al. (2015) concluded that assignment to donor populations was reliable even under various genetic drift scenarios. Correa and Moran (2017) estimated 10% of mis‐assignment of simulated hybrid genotypes to other nonparental populations. Our simulations suggest that misassignment of hybrid genotypes was much lower (0.5%), possibly because we simulated genotypes from parental geographic regions, not individual populations (Correa & Moran, 2017), or because SNPs and microsatellites differ in their assignment performance (Narum et al., 2008).

Both individual assignment and genetic mixtures supported a broad geographic origin of donor populations spanning six geographic regions, consistent with introduction records and the history of propagules imported to South America (see *Appendix* 1 for additional details) as well as a parallel study employing microsatellite markers on some of the same locations (Correa & Moran, 2017). Some uncertainty was associated with estimation of uncommon donor geographic regions in mixtures, reflected in wide 95% CIs, some of which contained zero as lower bound. A diverse origin of propagules contrasts strongly with the Chinook salmon gene pool in New Zealand, the most closely studied comparable case, where the species seems to have originated from a single source from California (USA), and dispersed largely by natural reproduction (McDowall, 1994; Quinn et al., 1996). The New Zealand scenario resembles *natural* populations in our study as they were composed of a single region, but located geographically in the *Lower Columbia River‐Willamette*. Populations tracking their origin to this dominant region likely originated from sea‐ranching experiments at PRA from 1982 to 1988; it was also the likely source of Chinook salmon invading Atlantic Ocean basins via dispersal (Ciancio et al., 2015; Riva‐Rossi et al., 2012). Interestingly, PRA received during the same period a significant number of propagules from the University of Washington broodstock (43.2%), members of the *Puget Sound‐South British Columbia* region.

What is the explanation behind the predominance of *Lower Columbia River‐Willamette* geographic region? Successful invasions are often a combination of stochastic and directional forces (Keller & Taylor, 2008). We speculate that genetic drift, selection on existing preadapted genes, or both, may be potential explanations. Selection on preadapted genes or the “preadaptation hypothesis” implies that specific populations will be successful only on specific environments (Chown et al., 2015; Sax & Brown, 2000), and Chinook salmon lineages may be no exception. Narum et al. (2017) compared native and non‐native Chinook salmon environments using high‐resolution global climate layers. They concluded that non‐native environments had higher precipitation and lower temperatures; they also found variation among Patagonian sites located at various latitudes (Narum et al., 2017). Thus, whether temperature, precipitation, flow regime, or migration distance from sea among South America basins have provided opportunities for selection of specific Chinook salmon lineages deserves further scrutiny by contrasting genetic and phenotypic data from both native and non‐native populations.

We found evidence for hybridization between non‐native Chinook salmon lineages as several individuals from *artificial* populations were assigned to two or more geographic regions. This was also reported by Correa and Moran (2017). It is unclear whether hybridization between lineages occurred in captivity prior to stocking, progressively in the wild following multiple stocking events or aquaculture escapes, or all the above. No significant departures from HWE proportions and linkage equilibrium within collections suggest that perhaps enough generations have passed as to dissipate Wahlund effects. However, whether admixture enhances invasiveness among non‐native populations is still unresolved (Hahn & Rieseberg, 2017; Rius & Darling, 2014; Wolfe, Blair, & Penna, 2007).

#### 4.3.2 Relative propagule pressure

Propagule pressure may help mitigate demographic, environmental, and genetic stochasticity among non‐native populations, and can thus be a key factor explaining why species become invasive (Simberloff, 2009). Although propagule pressure was shown to increase genetic diversity among *artificial* populations in this study and elsewhere (Consuegra et al., 2011; Roman & Darling, 2007), little is known about how relative propagule pressure may influence genetic mixtures. We found consistency between genetic mixtures and relative propagule pressure inferred from introduction records of Chinook salmon for two of three basins analyzed, Petrohué River (PET) and Prat River (PRA). These findings suggest that the more propagules were introduced from a specific geographic region, the larger its genetic contribution, in line with demographic effects of propagule pressure during the establishment phase of an invasion (Szűcs, Melbourne, Tuff, & Hufbauer, 2014). In the case of PET and PRA, most propagules originated from *Puget Sound‐South British Columbia*; however, *Lower Columbia River‐Willamette* became more represented in genetic mixtures in the case of PRA.

We additionally found that measures of relative propagule pressure and mixture analyses for Chinook salmon populating Cobarde River (COB) were inconsistent, suggesting that the demographic effects of propagule pressure cannot be generalized, and that history and chance or deterministic factors may affect invasion success (Keller & Taylor, 2008). Di Prinzio et al. (2015) used genetic mixture analysis to track donor geographic regions for Chinook salmon from Futaleufú River, located only 150 km south of PET, and found that the largest contribution came from *California Central Valley* not *Puget Sound‐South British Columbia* as for PET. This suggests different genetic outcomes for two adjacent basins stocked with Chinook salmon from similar donor populations. We hypothesize that propagules of *Puget Sound‐South British Columbia* origin may have initially established at COB, but failed to thrive in subsequent generations; they were possibly replaced by immigrants from southern populations (e.g., PRA) carrying genes from *Lower Columbia River‐Willamette* region.

## 5 CONCLUSION

*Artificial* and *natural* dispersal pathways left unique signals on genetic metrics, the evolution of genetic structure, and degree of admixture among non‐native Chinook salmon populations in South America. *Artificial* populations had higher genetic diversity and larger estimates of *N*_b_ than *natural* populations. Gene flow seemed more common between adjacent *artificial* and *natural*, or adjacent *natural*, than geographically distant populations. Findings were consistent with a process of colonization in which high‐diversity *artificial* populations likely established first followed by founding of low‐diversity *natural* populations. Genetic mixtures helped identifying donor geographic regions and the influence of relative propagule pressure. Overall, the study of non‐native Chinook salmon mixtures in combination with well‐documented introductions and their origin represents a unique approach to study differential invasion success among genetically distinct donor populations.

## DATA ACCESSIBILITY

SNP multilocus genotypes for this study are available in DRYAD under accession number doi: (https://doi.org/10.5061/dryad.5k45n83).

## CONFLICT OF INTEREST

None declared.

## AUTHOR CONTRIBUTIONS

DG‐U, CR‐R, JEC, MAP, JES, and LWS contribute to conception and design of the work; DG‐U, DC‐R, BE, EA, SSM, FV‐A, MAP, and TPQ contributed to acquisition, analysis, and interpretation of data for the work; DG‐U and DC‐R drafted the manuscript; DG‐U, CR‐R, JEC, TPQ, JES, and LWS revised the manuscript critically for intellectual content; and all authors approved the final version to be published and agree to be accountable for all aspects of the work in ensuring that questions related to the accuracy or integrity of any part of the work are appropriately investigated and resolved.

## APPENDIX 1

### 1.1

We discussed in further detail the origin of Chinook salmon introductions from introduction records and present‐day inferences of population mixtures. We matched evidence from these two sources among stocked (*artificial*) populations from our study and present additional details on how we assigned the hatchery of origin of Chinook salmon propagules (often eggs that were hatched and reared to juveniles before stocking) to geographic regions.

### ALLIPEN (ALP) RIVER

There were no official records of Chinook salmon stocking on this river and other tributaries from Toltén River basin. However, we found evidence for a company‐operated hatchery authorized by Chile's Undersecretariat of Fisheries (Res. Ex. 393/1993) to maintain broodstock of Chinook salmon. Broodstock was likely acquired from another hatchery located in the Lake District, presumably from the Sacramento River (geographic region: *California Central Valley*) during 1993–1995 in one of ALP's tributaries near the city of Melipeuco (Zubillaga, 2013). According to Zubillaga (2013), hatchery company owners released their broodstock to ALP in 1995 after filing for bankruptcy. Since 2000, large numbers of Chinook salmon adults became common on spawning grounds in several of ALP's tributaries. Strong genetic divergence between ALP and the rest of Chinook salmon populations located further south assessed from various methods supports the hypothesis of an independent, artificial introduction in the Toltén River basin. Evidence from genetic mixtures is nonetheless in conflict with anecdotical data regarding the hatchery of origin of Chinook salmon released to ALP. Most were assigned to *Oregon‐California Coast* (46%), followed by *Lower Columbia River—Willamette* (36%), and none to *California Central Valley* (Table 4). This is consistent with large numbers of eggs imported to Chile's Lake District from Oregon during 1990s (Table 5). All Oregon State hatcheries can be located within geographic region *Oregon‐California Coast* (Warheit et al., 2012).

### PETROHUE (PET) RIVER

One of the first basins to be stocked (1978–1994) and received the highest propagule number and size documented among Pacific Ocean basins. Propagule origin from mixture analysis and introduction records agree closely and span four geographic regions: *Lower Columbia River* via Cowlitz River and other hatcheries, *Puget Sound*—*South British Columbia* via University of Washington broodstock (Seattle), *Oregon‐California Coast* via Oregon, and *California Central Valley* via New Zealand (Correa & Gross, 2008; Donaldson & Joyner, 1983; Niklitschek & Toledo, 2011; Quinn et al., 1996; Riva‐Rossi et al., 2012). Genetic mixture analysis suggests contributions of both early sea‐ranching and late aquaculture broodstock from various geographic sources. They contributed with founding individuals to PET and nearby basins, such as Futaleufú River (Di Prinzio et al., 2015).

### ESTERO PICHICOLO (PIC)

It harbors one of the few Chinook salmon hatcheries in Chile. The origin of the broodstock was Washington State, most likely from University of Washington (Riva‐Rossi et al., 2012). Mixture analysis shows a large contribution from *Puget Sound—South British Columbia* (86%) which is consistent with this prediction. However, contributions from *Columbia River—Deschutes* (11%) and *California Central Valley* (3%) were also found, suggesting admixture with other geographic regions. Astorga, Valenzuela, Arismendi, and Iriarte (2008) found significant genetic divergence between PIC and PET using three microsatellite DNA markers, and argued that sources other than PIC were likely founders of Chinook salmon at PET. Individual‐based analyses in this study showed on the contrary that these two populations clustered within the same group and genetic differentiation was weak, albeit significant (θ = 0.03; Table S2). These findings imply that their origin is similar and can be tracked back to University of Washington broodstock, though escapes from PIC that may have contributed to PET cannot be ruled out.

### COBARDE (COB) RIVER

Historical records indicated that Chinook salmon broodstock used in net‐pen aquaculture near Magdalena Island and Aysén River basin were imported from Vancouver, British Columbia (Niklitschek & Toledo, 2011; Riva‐Rossi et al., 2012). The hatchery of origin was likely from Big Qualicum River, in operation since 1967 (MacKinlay, Lehmann, Bateman, & Cook, 2004), and located geographically within *Puget Sound—South British Columbia* geographic region. Surprisingly, mixture analyses showed that a small proportion of the population originated from this group (3%); instead, a massive genetic contribution came from *Lower Columbia River—Willamette* (96%). This suggests that sources other than net‐pen aquaculture may have provided Chinook salmon propagules for COB's population (see below).

### PRAT (PRA) RIVER

The second basin in South America to be successfully stocked (after PET) following sea‐ranching experiments in the Magallanes District during 1982–1989. Chinook salmon propagules originated from three geographic regions according to historical records: *Puget Sound—South British Columbia* (University of Washington broodstock, Green River hatchery in Puget Sound), *Lower Columbia River—Willamette* (broodstock from returning adults successfully stocked to the Lake District: see PET), and *Oregon‐California Coast* (from Oregon hatcheries). Genetic mixture analyses were consistent with the two former groups, but found no evidence of the third group, *Oregon‐California Coast*, consistent with the lowest propagule pressure (Table 5). Chinook salmon from PRA showed equal genetic membership to *Puget Sound—South British Columbia* and *Lower Columbia River—Willamette*, which explains why some individuals clustered with PET, COB‐VAR‐SER, or where intermediate (Figure 3).
