# Supplementary figures and images for: Genetic signals of artificial and natural dispersal linked to colonization of South America by non‐native Chinook salmon (Oncorhynchus tshawytscha)
Source: Ecol Evol. 2018 May 24;8(12):6192–209. doi: 10.1002/ece3.4036 (PMC6024130; doi:10.1002/ece3.4036)

**F1**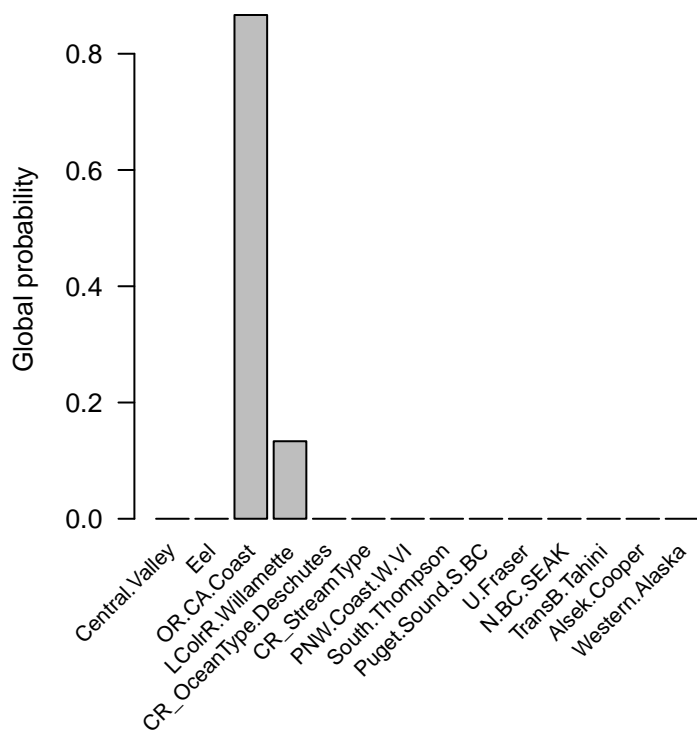**F2**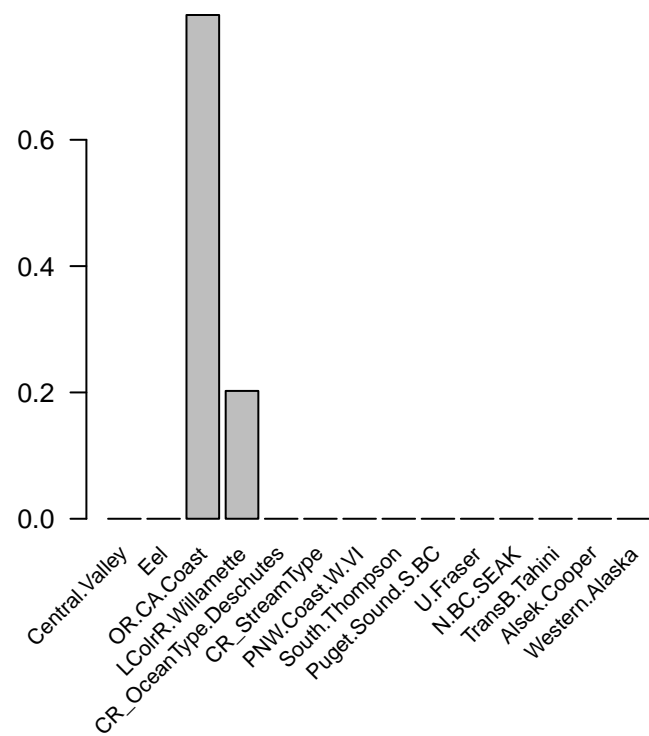**BC F1 and LCol**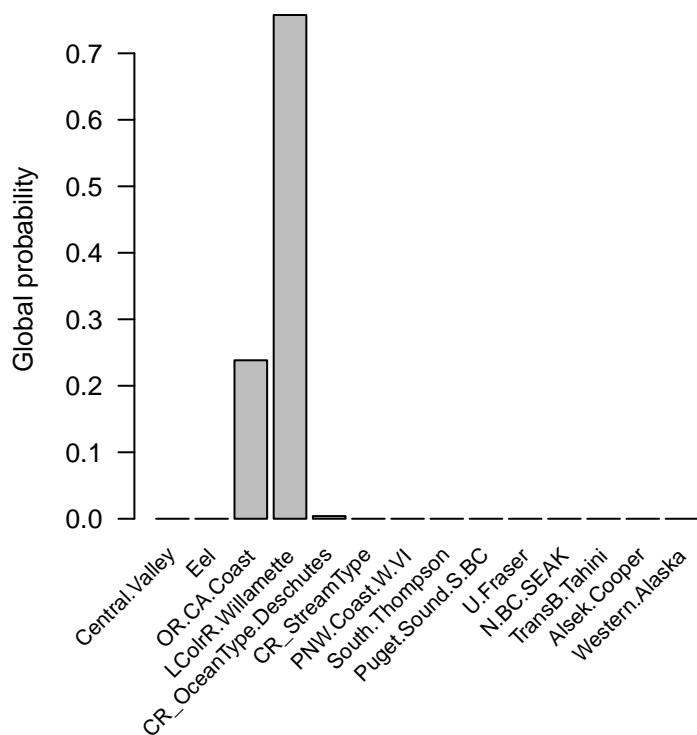**BC F1 and OrCa**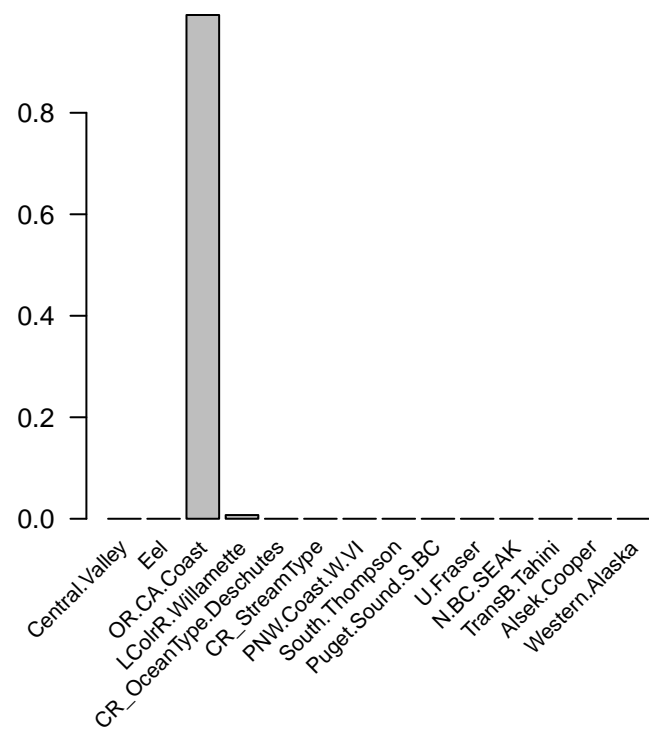

Supplement: Supplementary file 1 [file ECE3-8-6192-s001.pdf]

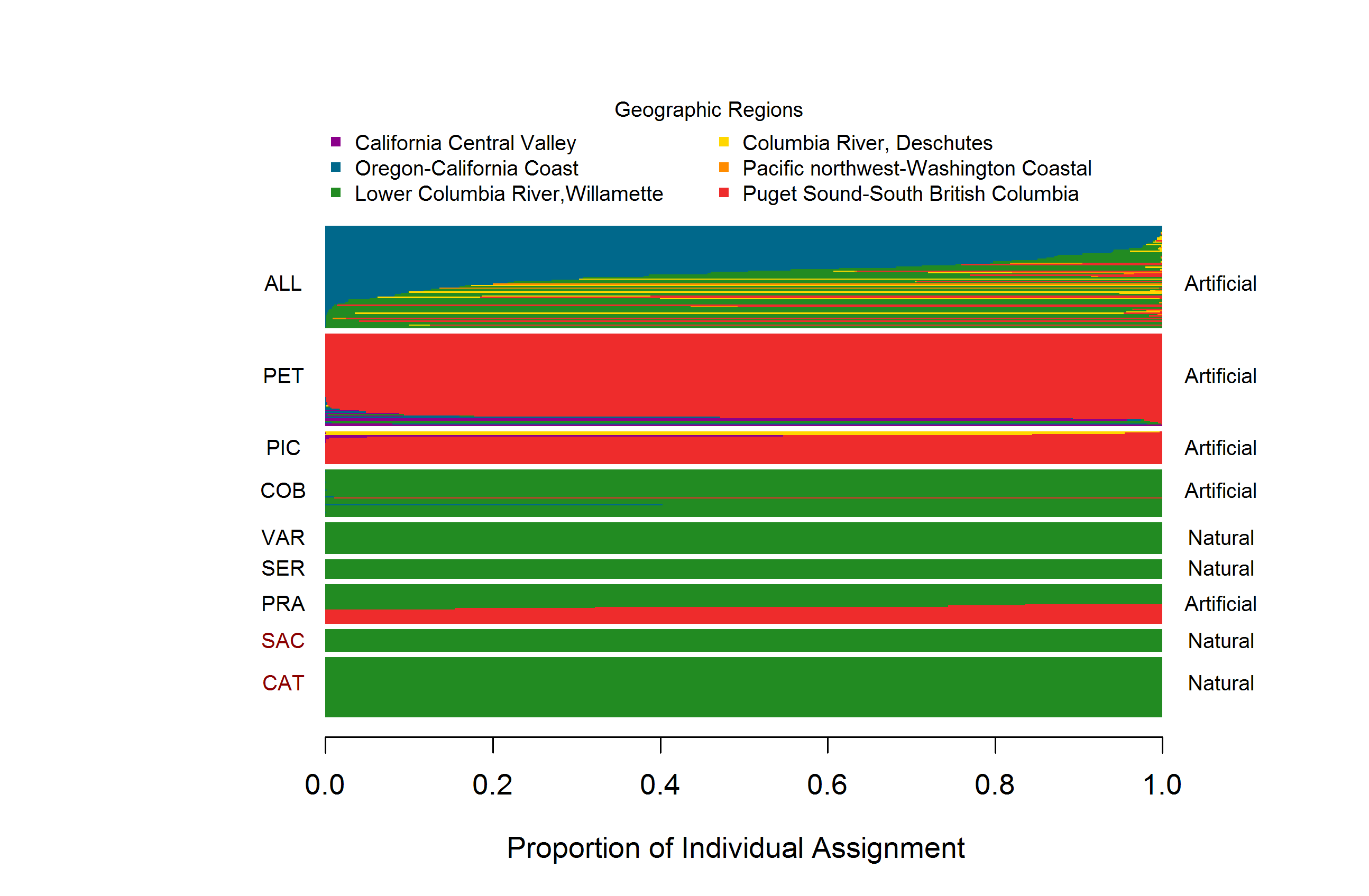

Supplement: Supplementary file 2 [file ECE3-8-6192-s002.tiff]
